# Pancreatic Exocrine Insufficiency in Pancreatic Cancer

**DOI:** 10.3390/nu9030183

**Published:** 2017-02-23

**Authors:** Miroslav Vujasinovic, Roberto Valente, Marco Del Chiaro, Johan Permert, J.-Matthias Löhr

**Affiliations:** Center for Digestive Diseases, Karolinska University Hospital, Stockholm SE-141 86, Sweden; miroslav.vujasinovic@karolinska.se (M.V.); roberto.valente@karolinska.se (R.V.); marco.del-chiaro@ki.se (M.D.C.); johan.permert@karolinska.se (J.P.)

**Keywords:** pancreatic exocrine insufficiency, pancreatic cancer, pancreatic surgery, malnutrition, vitamin

## Abstract

**Abstract**: Cancer patients experience weight loss for a variety of reasons, commencing with the tumor’s metabolism (Warburg effect) and proceeding via cachexia to loss of appetite. In pancreatic cancer, several other factors are involved, including a loss of appetite with a particular aversion to meat and the incapacity of the pancreatic gland to function normally when a tumor is present in the pancreatic head. Pancreatic exocrine insufficiency is characterized by a deficiency of the enzymes secreted from the pancreas due to the obstructive tumor, resulting in maldigestion. This, in turn, contributes to malnutrition, specifically a lack of fat-soluble vitamins, antioxidants, and other micronutrients. Patients with pancreatic cancer and pancreatic exocrine insufficiency have, overall, an extremely poor prognosis with regard to surgical outcome and overall survival. Therefore, it is crucial to be aware of the mechanisms involved in the disease, to be able to diagnose pancreatic exocrine insufficiency early on, and to treat malnutrition appropriately, for example, with pancreatic enzymes.

## 1. Introduction

Every cancer induces weight loss and eventually cachexia through different mechanisms but when cancer occurs in the pancreas, the central organ for digestion, this phenomenon is amplified with devastating metabolic consequences. The reason being that the pancreas produces pancreatic juice, whose activity in the intestinal lumen plays a major role in the digestive process. 

Pancreatic juice consists of a mixture of bicarbonates and water (secreted by the ductal component of the pancreas) and several enzymes (secreted by the acinar component) that are involved in the digestion of nutrients, particularly carbohydrates, proteins, and fat [1]. Pancreatic enzyme secretion has three phases: cephalic, gastric, and intestinal. The first two are mediated by neuro-vagal nerve stimulation while the third, and probably the most important, is regulated by the release of the hormones cholecystokinin (CCK) and secretin from the duodenal wall (stimulating acinar cells and ductal cells to produce enzymes and the water-bicarbonate mix, respectively) [2,3]. The main trigger for the third phase is the presence of fatty acids, amino acids, and gastric acid in the duodenum [4]. 

Digestion is a complex process that requires the simultaneous presence of both pancreatic enzymes and food in the duodenal lumen [1]. Therefore, conditions that lead to maldigestion can be either organic (impaired secretion of pancreatic enzymes), functional (impaired coordination between enzymes and food), or a combination of the two conditions. 

Pancreatic exocrine insufficiency (PEI) is defined as the condition in which the amount of secreted pancreatic enzymes is insufficient to maintain normal digestion [5], while pancreatic cancer is a typical condition in which normal pancreatic exocrine secretion is impaired due to chronic obstructive damage to the secreting component of the organ. 

Pancreatic surgery to remove the obstruction can cause even more complex digestive alterations influencing many aspects of the digestive process. In fact, in this setting, modifications of gastrointestinal anatomy, progressive functional changes caused by the underlying pancreatic disease, the extent of pancreatic tissue removed, reduced postprandial stimulation, and asynchrony between gastric emptying of nutrients and pancreatic enzyme secretion, all play a major role in the establishment of severe maldigestion [6]. 

The most frequently described sign of PEI is steatorrhea, which is defined as a stool fat content of >7 g/day (when the diet contains 100 g of fat) with associated symptoms of abdominal pain, flatulence, and weight loss [7]. Nevertheless, it is noteworthy that the malabsorption of fat generally does not occur until pancreatic lipase and trypsin levels fall to below 5%–10% of normal production. Table 1 summarizes the different mechanisms underlying intraluminal pancreatic enzyme deficiency (Table 1) [7,8,9,10].

Neoplasms of the pancreas may originate from both exocrine and endocrine cells. Ductal adenocarcinoma and its variants make up more than 90% of all malignant exocrine pancreatic tumors, while pancreatic endocrine tumors display a low incidence (1%) but a considerable prevalence (around 10% of all pancreatic masses) [9]. Rarer tumors include cell acinar carcinomas, sarcomas, and tumors of uncertain histogenesis, which account for a minority of cases [10]. 

Of the exocrine tumors, those occurring in the head of the pancreas, are more common and are generally associated with a high level of PEI, possibly related to the obstructive effect on the ductal system caused by tumor growth [10,11]. 

## 2. Pathophysiological Considerations

**Figure 1:**
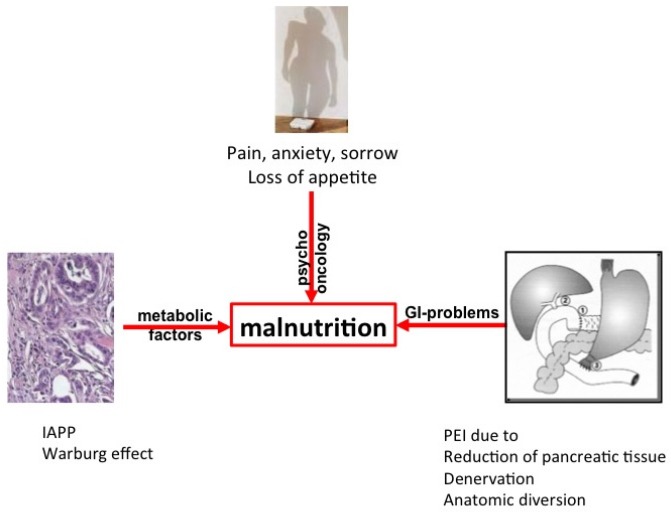
Intrinsic (tumor cell-triggered) and extrinsic (host-triggered) factors contributing to malnutrition in pancreatic cancer patients. For details and references see text. IAPP = islet amyloid polypeptide; PEI = pancreatic exocrine insufficiency. GI = gastrointestinal

The development of a tumor is a complex process, starting with the clonal expansion of atypical cells that can no longer be recognized by the defense mechanisms of the organism [12]. Later in the process (Figure 1), energy expenditure leads to wasting as the tumor’s metabolism draws energy from the body (known as the Warburg effect) via cachexia to loss of appetite [13,14]. In pancreatic cancer, several additional factors are involved, including a loss of appetite with a particular aversion to meat [15] (Table 2). Furthermore, tumor-derived factors such as islet amyloid polypeptide (IAPP) contribute to both cachexia and the loss of appetite [16,17]. A silent, subclinical (smoldering) inflammation with increased C-reactive protein (CRP), present in many solid tumors and well described in pancreatic cancer [18], also contributes, to a certain degree, to both energy expenditure and loss of appetite; indeed, elevated CRP levels can be used as a marker for cachexia in pancreatic cancer [19] and can even predict a poor prognosis [20]. Finally, as most of the tumors occur in the head of the pancreas, tumor growth results in the obstruction of the main pancreatic duct and the subsequent (complete) reduction of the secretion of pancreatic enzymes during meals. This gives rise to malnutrition secondary to maldigestion due to PEI and subsequent weight loss. As a result, some patients with pancreatic cancer have already undergone significant weight loss by the time the disease is diagnosed [21] and it is these patients who have the worst prognosis [22].

## 3. Historical Perspectives

The first anatomical resection of a solid pancreatic tumor was a distal pancreatectomy (DP), performed by Friedrich Trendelenburg in 1882 in Germany [23], while the first successful partial pancreaticoduodenectomy (PD) in patients with periampullary cancer was performed as a two-stage operation by the German surgeon Walther Kausch in 1909. In 1934, in the USA, Allen Whipple performed the first anatomical PD for an ampullary carcinoma, which he perfected to a one-stage resection by 1940 [10,23]. This operation was known as a pancreaticoduodenectomy, or Whipple resection, and involved a partial gastrectomy (antrectomy), cholecystectomy, and removal of the distal common bile duct, the head of the pancreas, duodenum, proximal jejunum, and regional lymph nodes. Reconstruction requires a pancreaticojejunostomy, hepaticojejunostomy, and a gastrojejunostomy [10]. The Whipple procedure remained the gold standard resection technique for cancers involving the head of the pancreas until Traverso and Longmire reintroduced the concept of pylorus preserving pancreaticoduodenectomy (PPPD) in 1978 to reduce the incidence of postgastrectomy syndrome and marginal ulceration [23,24]. During the 1990s, Japanese surgeons advocated the use of more radical pancreatic resections to improve cure rates (radical extended Whipple resection) [10]. The standard Whipple procedure was modified by the removal of more peripancreatic soft tissue and the lymph nodes, often with resection of segments of the superior mesenteric and portal veins, when they appear to be involved with the tumor [10,25].

Although pancreatic resection for tumors is the only chance of long-term survival for patients with pancreatic cancer, this procedure is not without immediate surgical risk or long-term sequelae [7,26]. This applies in particular to the patient’s nutritional situation. The extensive resection and reconstruction of the upper gastro-intestinal tract disrupts the physiological process of digestion and can trigger PEI.

## 4. Diagnosing Pancreatic Exocrine Insufficiency

There are several tests, both indirect and direct, available for assessing PEI [27]. Measuring fecal elastase-1 (FE-1) is a very simple, indirect, and non-invasive method of evaluating pancreatic enzyme secretion. FE-1 is produced exclusively by the pancreas and is not affected by breakdown in the intestine, and thus provides a direct representation of the secretory capacity of the gland. It is widely accepted that the lower the FE-1 concentration, the higher the probability of PEI. However, the FE-1 test is not capable of excluding mild to moderate PEI, and there is no consensus regarding the cut-off for PEI; although a threshold of 200 µg/g has been used most frequently [8]. The coefficient of fat absorption (CFA) is generally accepted as the gold standard for the diagnosis of steatorrhea, which is characteristic of severe PEI, but it is rarely used due to its limitations in terms of specificity, availability, patients’ compliance, and handling of fecal samples in the laboratory [28]. The ^13^C mixed triglyceride breath test (13C-MTG-BT) is an alternative diagnostic method that measures the resulting malnutrition rather than pancreatic enzyme secretion [29]. However, it is not yet widely available and has only been commercialized in some European countries [28].

## 5. Pancreatic Exocrine Insufficiency in Patients with Pancreatic Tumors

In 2000, Ong et al. reported the first retrospective study on the incidence of PEI after PD and pancreaticogastrostomy were performed in 11 patients suffering from pancreatic ductal adenocarcinoma (PDAC), duodenal cancer, ampullary cancer, cholangiocarcinoma, and duodenal leiomyoma (Table 3) [30]. The patients in the study group had significant PEI (diagnosed by fecal chymotrypsin) compared to the control group of 11 consecutive patients who had undergone subtotal gastrectomy (SG) for distal stomach tumors.

In 2002, Armstrong et al. performed the first prospective study using FE-1 as a diagnostic tool. The study included 10 patients who had undergone PD for malignant periampullary tumors and pancreatic exocrine function was evaluated at least six months after the operation [31]. The FE-1 measurements suggested that all patients had severe exocrine insufficiency (with six patients having a measured FE-1 level <15 µg/g). In addition, dietary deficiencies of fat-soluble vitamins and trace elements (vitamins D, A, E, zinc, selenium, iron) were also detected. 

Matsumoto and Traverso retrospectively analyzed data on 138 patients in whom PD was performed by the same surgeon [32]. The PD procedure was pylorus-preserving in 94% of patients and preoperative FE-1 values were normal in 78%. When compared with preoperative values, the percent of cases with reduced FE-1 levels at three months, one year, and two years postoperatively was 48%, 73%, and 50%, respectively. 

In another study, Spiecher and Traverso analyzed 83 patients after PD in whom FE-1 was measured pre- and post-operatively [34]. At 24 months after surgery, all patients with normal pre-operative FE-1 values had maintained their normal exocrine status. Of the patients whose resection was limited to the left of the portal vein, all patients had normal postoperative FE-1 values at three, 12, and 24 months. Of those whose resection extended over or beyond the portal vein, 88% had normal postoperative FE-1 values at three months and 100% were normal at 24 months. However, the differences between this study in comparison to those mentioned previously were probably related to patient selection criteria, since 55% of patients had cystic neoplasms (serous cystadenoma, intraductal papillary neoplasm, or mucinous cystadenoma) while only 12% of patients suffered from PDAC. 

In a prospective study, Halloran et al. examined the symptoms, CFA, quality of life (QoL), and the accuracy of the FE-1 measurement in predicting PEI in 40 patients who had undergone partial pancreatic resection for pancreatic malignancy [7]. PEI was present and sustained in a majority of patients following resection and was not associated with particular symptoms. However, these patients had a tendency towards poorer QoL values. A comparison of FE-1 using a cut-off point of 200 µg/g for PEI against CFA showed a diagnostic accuracy of 70%. Further testing in the same study showed that the optimal cut-off for FE-1 (to diagnose PEI as defined by CFA <93%) was in fact lower, at 128 µg/g (sensitivity: 90%, specificity: 44%).

QoL was also determined in another study performed by Belyaev et al. [36]. In a cohort of 174 patients, patient age and the development of postoperative endocrine and exocrine pancreatic insufficiency were relevant prognostic factors. These significantly affected the postoperative QoL, mainly the physical health of patients.

In the biggest prospective study performed so far, Partelli et al. demonstrated, for the first time, a correlation between a low FE-1 value and poor survival in patients with advanced PDAC, with an FE-1 value ≤20 µg/g as an independent predictor of survival. The median overall survival was significantly higher in patients who had an FE-1 >20 µg/g (11 months) compared to those with FE-1 ≤20 µg/g (7 months) [35].

Tran et al. assessed the correlation between preoperative changes in the pancreatic parenchyma and postoperative exocrine and endocrine function at least six months after PD [8]. Of the 74 patients, 56 (76%) had FE-1 levels <100 µg/g, indicating severe pancreatic insufficiency and 9 patients had FE-1 levels between 100 and 200 µg/g, indicating mild insufficiency. The authors concluded that the main reasons for the development of insufficiency were fibrosis and loss of functional tissue. The mechanisms underlying exocrine function in pancreatic cancer have not been elucidated fully and the risk of patients developing exocrine insufficiency has not been well established.

Sikkens et al. assessed the effect of pancreatic cancer resection on exocrine function in patients on a monthly basis from the time of diagnosis. To preclude the confounding effects of a pancreatic resection, they only evaluated inoperable patients [37]. Exocrine insufficiency was present at the time of diagnosis in 21/32 (66%) patients and at the end of follow-up in 22/24 (92%) patients.

Japanese authors have reported a high incidence of PEI diagnosed using the 13C-MTG-BT [38,39,40]. Most of the studies performed so far have shown clearly that the incidence of PEI after PD for pancreatic tumors is high, with a range of 64%–100%. The incidence of PEI after DP is lower than after PD, and is within a range of 0%–42%. However, there is a lack of well-designed studies on PEI in patients with pancreatic tumors, and of these, most are retrospective [30,32,33,34,36], include different types of surgery and/or have small sample sizes [7,30,31,33,37], are single-center designed and include a heterogeneous patient population with both benign and malignant disease [32,34,36]. Thus, there is an urgent need for high volume, well-designed, prospective, multi-centered studies with pre- and post-operative determination of patients’ clinical and laboratory data.

## 6. Pancreatic Enzyme Replacement Therapy (PERT)

PEI leads to malabsorption, which causes steatorrhea, weight loss, and malnutrition. It is associated with deficiencies of the fat-soluble vitamins, trace elements, and essential fatty and amino acids [41]. PEI may be subclinical or associated with two types of symptoms: those associated with the presence of undigested food within the intestinal lumen (causing fatty diarrhea, flatulence, and dyspeptic symptoms) and those associated with nutrient loss (mainly resulting in weight loss and fat-soluble vitamin deficiency) [6].

Unfortunately, PEI is frequently overlooked in daily clinical practice, and physicians are often not well informed about the need to supplement PEI. This was confirmed in a survey conducted on members of the Dutch and German patient associations for pancreatic disorders, that clearly showed that most patients suffering from PEI after pancreatic surgery are undertreated even in countries with well-organized health care systems [41]. In an Australian retrospective study on 129 patients with metastatic pancreatic cancer and symptoms that could be attributable to malabsorption, only 21% patients were prescribed PERT [42]. Another study highlighted a clear clinical gap in managing PEI in patients with supportive care for pancreatic cancer [43]. The findings revealed that the major QoL issue was difficulty in managing gut symptoms and complex dietary issues due to a lack of information about malabsorption and PEI (lack of routine dietary consultation, perceived reluctance of clinicians to prescribe enzyme supplements and poor understanding of dose-to-diet guidelines). The effect of PERT was also evaluated in patients with unresectable pancreatic cancer. Sixty-seven patients were randomized to receive either enteric-coated PERT or a placebo [44]. Intent-to-treat analysis showed no significant difference in the percentage change in body weight at eight weeks between the PERT and placebo groups.

However, in the context of new oncologic protocols that improve the survival of patients with pancreatic cancer, it is vital to optimize the performance status of patients with pancreatic cancer in order to make those patients eligible for new adjuvant or palliative options in the future [45].

FE-1 should be performed routinely, even in asymptomatic patients, since clinical signs of steatorrhea may be absent in patients that tend to limit fat intake to reduce their symptoms. Pancreatic enzyme supplementation should be considered in every patient with proven PEI, irrespective of the underlying disease or prognosis. Even in the case of asymptomatic exocrine insufficiency, treatment is indicated because studies have shown that malnutrition may develop in these patients [41]. Compared with untreated patients, patients with advanced pancreatic cancer who are given pancreatic enzyme supplements enjoy a better QoL and improved symptom score [46]. Current guidelines strongly advocate the use of PERT in patients with pancreatic cancer to maintain weight and increase QoL [47]. To reduce morbidity and mortality, it is very important to treat patients with a sufficient dose of pancreatic enzymes and PERT should be initiated once PEI is diagnosed or even when there is a strong clinical suspicion of PEI [6]. Two double-blind randomized controlled trials evaluated PERT for PEI after pancreatic surgery [48,49]. In these studies, when compared with a placebo, PERT (in the form of pancreatin formulated as enteric-coated minimicrospheres) was associated with a significant improvement in fat and protein digestion after pancreatic resection for chronic pancreatitis or pancreatic cancer. In addition, PERT was associated with significant weight gain and reduced stool frequency. Enzyme doses of 72,000–75,000 Ph.U. of lipase with main meals and 36,000–50,000 Ph.U. with snacks have shown to be effective in terms of improvement in fat digestion [48,49].

## 7. Conclusions

The factors contributing to malnutrition in pancreatic cancer are manifold; however, PEI stands out as a major contributor, in both inoperable patients and operable patients. Pancreatic surgery is frequently associated, not just with immediate surgical complications, but also with long-term sequelae. Most of the studies performed so far have shown clearly that the incidence of PEI after pancreatic surgery for tumors is high. Unfortunately, PEI is also an under-recognized and under-treated complication of pancreatic surgery. FE-1 should be performed routinely at the time of diagnosis of a pancreatic tumor and during post-operative follow-up; as well as in asymptomatic patients, because clinical signs of steatorrhea may be absent as patients tend to limit fat intake to reduce symptoms. PERT should be considered in every patient with proven PEI, irrespective of the underlying disease or prognosis and treatment is indicated even in the case of asymptomatic exocrine insufficiency, as malnutrition may develop in these patients. The enzyme dose of PERT needs to be individually adjusted based on the patient’s clinical and nutritional parameters, residual pancreatic function, and dietary fat intake. Patients suffering from pancreatic cancer and PEI generally have an extremely poor prognosis and thus it is crucial to be aware of the disease mechanisms to be able to diagnose PEI early on, and to treat malnutrition appropriately.

## Figures and Tables

**Table 1:** Factors contributing to pancreatic exocrine insufficiency.

Primary PEI* (Intrinsic/Pancreatic)	Secondary PEI (Extrinsic/Intestinal)
Pancreatic fibrosis/chronic pancreatitis	Intestinal motility
Replacement of healthy pancreatic tissue with tumor Reduction of pancreatic tissue (surgery)	Low intestinal pH (peptic ulcer)
Diabetes mellitus (pancreatic exocrine atrophy)	Anatomic alteration (surgery)
Pancreatic duct obstruction	Stimulation/denervation (surgery, drugs, diabetes)

Compiled from [5] and [8]. PEI* = Peancreatic Exocrine Insufficiency.

**Table 2:** Factors contributing to weight loss and malnutrition in pancreatic cancer.

	Tumor-Triggered	Host-Triggered
Calorie demand	IAPP*/Warburg effect	⇧ at rest, ⇩ in total
		Food aversion (meat)
Exercise		Low
Psycho-oncological factors		Pain, anxiety, sorrow
Gastrointestinal factors		Small intestinal bacterial overgrowth

IAPP* = islet amyloid polypeptide. ⇧ = increased; ⇩ = descreased.

**Table 3:** Studies of pancreatic function in patients undergoing pancreatic surgery.

Study	Year	*N*	Patients Included	Diagnosis PEI	Type of Surgery	PEI
Ong [30]	2000	11	pancreatic ductal adenocarcinoma, duodenal cancer, ampullary cancer, cholangiocarcinoma, duodenal leiomyoma	fecal chymotrypsin	PD in all patients	36%
Armstrong [31]	2002	10	pancreatic ductal adenocarcinoma, duodenal cancer, ampullary cancer, cystadenocarcinoma, carcinoid tumor	fecal elastase-1 and NBT PABA test	PD in all patients	80% tested with NBT PABA and 100% tested with FE-1
Matsumoto [32]	2006	138	pancreatic ductal adenocarcinoma, periampullary cancer, IPMN, islet cell cancer, serous cystadenoma, mucinous cystadenoma, chronic pancreatitis	fecal elastase-1	PD in all patients	55%
Tran [33]	2008	55	pancreatic or periampullary carcinoma	fecal elastase-1	PD in all patients	87.8%
Speicher [34]	2010	83	pancreatic ductal adenocarcinoma, IPMN, islet cell tumor, serous cystadenoma, mucinous cystadenoma, chronic pancreatitis	fecal elastase-1	DP in all patients	30% in patients with pancreatical ductal adenocarcinoma prior to operation
Halloran [7]	2011	40	pancreatic ductal adenocarcinoma, periampullary cancer, cholangiocarcinoma, neuroendocrine tumor	CFA and fecal elastase-1	PD in 37 patients and DP in 3 patients	67% tested with CFA and 77% tested with FE-1
Partelli [35]	2012	194	advanced pancreatic ductal adenocarcinoma	fecal elastase-1	none	50%
Belyaev [36]	2013	104	malignant tumors, benign tumors, chronic pancreatitis	fecal elastase-1	PD in 49 patients, DP in 20 patients, TP in 19 patients	90.2%
Sikkens [37]	2014	29	pancreatic ductal adenocarcinoma, ampullary cancer, cholangiocarcinoma	fecal elastase-1	PD in 26 patients and DP in 3 patients	92%

*N* = number of patients included in the study; PEI = pancreatic exocrine insufficiency; NBT PABA = *N*-benzoyl-l-tyrosyl-p-aminobenzoic acid; CFA = coefficient of fat absorption test; FE-1 = fecal elastase-1; IPMN = intraductal papillary mucinous neoplasia; PD = pancreatoduodenectomy; DP = distal pancreatectomy; TP = total pancreatectomy.
